# Cholesterol-Lowering Strategies for Cardiovascular Disease Prevention: The Importance of Intensive Treatment and the Simplification of Medical Therapy

**DOI:** 10.3390/jcm13071882

**Published:** 2024-03-25

**Authors:** Vincenzo Sucato, Antonella Ortello, Francesco Comparato, Giuseppina Novo, Alfredo Ruggero Galassi

**Affiliations:** Division of Cardiology, Department of Health Promotion, Mother and Child Care, Internal Medicine and Medical Specialties (PROMISE) “G. D’Alessandro”, University Hospital Paolo Giaccone, University of Palermo, 90133 Palermo, Italy; antonellaortello@gmail.com (A.O.); giuseppina.novo@unipa.it (G.N.);

**Keywords:** cardiovascular prevention, lipid-lowering therapy, polypill, statin, ezetimibe, PCSK9 monoclonal antibodies, bempedoic acid, inclisiran

## Abstract

Cardiovascular diseases (CVDs) are a leading global cause of mortality and are primarily driven by atherosclerotic coronary artery disease. Their pathogenesis involves multi-factorial mechanisms, among which low-density lipoprotein (LDL) plays a causative role. Recent ESC/EAS guidelines advocate for a shift toward new risk estimation algorithms that better emphasize non-fatal cardiovascular events, lifetime risk prediction, and tailored pharmacological approaches, including statin + ezetimibe and triple therapy, in specific cases. Intensive lipid-lowering therapy has been shown to be pivotal, especially in post-acute coronary events. Intracoronary imaging has revealed insights into the composition of plaque and demonstrated the significant regression that can be achieved through the use of statins such as rosuvastatin and atorvastatin. The positive effects of Proprotein Convertase Subtilisin/Kexin type 9 (PCSK9) inhibitors, particularly alirocumab and evolocumab, on plaque regression, have been demonstrated. Inclisiran, which targets PCSK9 gene expression, significantly reduces LDL cholesterol. The associated challenges include hesitancy to prescribe intensive regimens and limited treatment adherence, highlighting the need for pharmacological combinations to improve therapeutic outcomes.

## 1. Introduction

Cardiovascular diseases (CVDs) represent one of the leading causes of mortality worldwide. According to the World Health Organization (WHO), approximately 32% of deaths worldwide in 2019 were attributed to cardiovascular diseases [1]. Among these diseases, atherosclerotic coronary artery disease plays a predominant role, standing out as the foremost cause of mortality and morbidity in the general population. Autopsy and in vivo studies have revealed atherosclerotic plaque to be the pathological substrate underlying the cascade of events culminating in coronary syndromes [2]. It is now well established that there are relationships between the extent of the disease, its rate of progression, and cardiovascular events [3,4].

The pathogenesis of atherosclerotic coronary artery disease is multi-factorial, but data from clinical and epidemiological studies have confirmed the causative role of low-density lipoproteins (LDLs) in the genesis and progression of the disease [5,6]. This has spurred research on pharmacological treatments aimed at controlling and reducing the atherosclerotic burden in patients at risk of developing coronary artery disease. A reduction in LDL levels is associated with a proportional decrease in the risk of cardiovascular events, both in terms of the extent of reduction and the duration of treatment [7]. This “lower is better” concept has prompted recent guidelines to substantially lower the LDL threshold [8]. Moreover, several studies have observed that reducing LDL levels below the targets recommended in the ESC/EAS guidelines is associated with a lower incidence of cardiovascular events [9,10,11,12]. Therefore, it is advisable to reduce LDL levels as much as possible, especially in patients characterized by very high cardiovascular risk.

The timing of treatment initiation also plays a crucial role. Various studies have shown that adopting intensive lipid-lowering therapy within 10 days of an acute coronary event reduces the risk to a greater extent when compared to non-intensive regimens [13,14]. In this regard, it is important not only to achieve therapeutic targets based on risk profiles and preventive measures but also to implement the most intensive therapeutic regimen possible.

## 2. Risk Stratification

The most significant modification to the 2021 ESC/EAS guidelines on cardiovascular prevention [15] is the recommendation of the use of new algorithms for risk estimation, replacing the previous SCORE system. Specifically, new calculators named SCORE2 (Systematic Coronary Risk Estimation 2) and SCORE2-OP (Systematic Coronary Risk Estimation 2—Older Persons) have been introduced for individuals aged over 70, along with the inclusion of non-fatal cardiovascular events (myocardial infarction and stroke) in the overall risk assessment (Table 1). In contrast to SCORE, in the risk tables developed using SCORE2, Italy is no longer classified as a low-cardiovascular-risk country but is categorized as having a moderate risk of cardiovascular mortality. This shift can be attributed to the inadequate implementation of cardiovascular prevention measures—especially in primary prevention—and the lack of attention to certain conditions, such as obesity, which are prevalent and prognostically significant.

Another noteworthy introduction is the concept of “lifetime risk”, which has become part of the decision-making and informational process for the patient. It involves predicting the age at which an individual has a 50% chance of developing a fatal or non-fatal cardiovascular event. Lifetime cardiovascular risk is determined using clinical experience and criteria such as age, risk factor levels (and their variations), and risk modifiers. It can also be estimated using specific risk score calculation methods.

A secondary benefit of a lifetime approach to managing and treating risk factors is represented by the numerical difference between the expected age at which there is a 50% probability of a person developing a cardiovascular event (in the absence of preventive interventions) and the estimated age with risk factor management (e.g., smoking cessation, cholesterol reduction, blood pressure control). These calculators are easily accessible online (e.g., the ESC CVD Risk Calculation app) and can be used for the estimation of cardiovascular risk and average lifetime benefit.

The latest 2021 ESC/EAS guidelines on cardiovascular prevention [15] recommend a systematic evaluation of cardiovascular risk in specific patient categories, namely:

- Apparently healthy individuals without confirmed atherosclerotic cardiovascular disease (ASCVD), diabetes mellitus, renal insufficiency, or familial hypercholesterolemia. The age groups for assessment are 55–75 years in women and 40–65 years in men, exhibiting a 10-year MCV risk variable around commonly used thresholds for intervention;
- Patients with chronic kidney disease in the absence of diabetes or ASCVD;
- Patients with familial hypercholesterolemia;
- Patients with diabetes mellitus, with or without organ damage, and possibly associated with known ASCVD. This category may also include patients with type 1 diabetes over the age of 40;
- Patients with clinically confirmed ASCVD (myocardial infarction, unstable angina, coronary revascularization, stroke, transient ischemic attack, aortic aneurysm, and peripheral arterial disease) or those detected through imaging tests as showing plaques on coronary angiography, carotid ultrasound, or computed tomography angiography (CTA). However, it does not encompass any increase in continuous variables obtained through imaging, such as the mean intima–media thickness of the carotid artery.

In the definition of risk, clinical and laboratory parameters are considered, the analysis of which allows for the determination of a specific risk class. The latest 2023 ESC/EAS guidelines for the treatment of cardiovascular diseases in diabetic patients [16] have brought about some innovations regarding risk stratification in this patient cohort. The previous prevention guidelines suggested using the ADVANCE score (Action in Diabetes and Vascular Disease: Preterax and Diamicron MR Controlled Evaluation); however, this score is less applicable to contemporary European populations. To address these limitations, the latest 2023 ESC/EAS guidelines recommend the use of SCORE2-Diabetes, an extension of the re-calibrated European SCORE2 system that is specifically applicable to individuals with type 2 diabetes aged 40–69 without ASCVD or severe organ damage, in order to estimate the individual 10-year risk of fatal and non-fatal cardiovascular events.

For each of the categories described above, different risk profiles (i.e., low–moderate, high, and very high) have been identified based on the probability of developing fatal and non-fatal cardiovascular diseases over 10 years, allowing for the identification of blood pressure and LDL cholesterol targets (Table 2).

The pharmacological approach utilizing currently available lipid-lowering drugs involves a stepwise strategy to achieve the defined LDL target, based on the designated risk category. The same guidelines [15] provide the absolute reductions in LDL levels that can be achieved with different therapeutic approaches (Table 3): the estimated average LDL level reduction ranges from 30% with moderate-intensity statin therapy to 50% with high-intensity statin therapy, and up to 65% with the addition of ezetimibe. Adding a PCSK9 inhibitor to high-intensity statin therapy alone or combined with ezetimibe can lead to LDL level reductions of 75% and 85%, respectively. The effect achieved through the use of a high-intensity statin can be achieved through administering moderate-intensity statins + ezetimibe. Recent evidence has supported the choice to improve therapeutic adherence through reducing the side-effects associated with the use of high-intensity statins [17].

**Figure 1:**
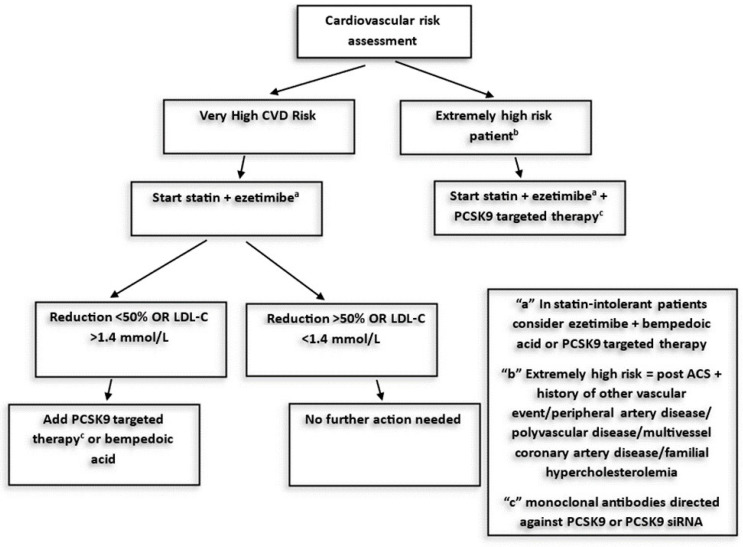
Strategies for LDL cholesterol reduction: combination therapy [19].

It is clear from the abovementioned information that new intervention strategies should be based not only on achieving the lowest LDL level but also on the early initiation of treatment, especially in the presence of specific conditions and clinical phenotypes [18]. Particularly in patients at very high risk, starting lipid-lowering therapy directly with the statin + ezetimibe combination could be advantageous. Similarly, in patients with extremely high risk (e.g., those with a recurrent event within 2 years), the use of triple therapy (statins + ezetimibe + PCSK9 inhibitors) before discharge could maximize the benefits of early combination therapy (Figure 1) [19].

For patients experiencing acute coronary syndrome (SCA) while on statin therapy, the first consideration should be the current drug and dosage: for those with a history of confirmed statin intolerance and SCA, prescribing ezetimibe 10 mg/day and a PCSK9 inhibitor is reasonable. Additionally, if the patient has elevated baseline LDL cholesterol levels (>150 mg/dL), then it is reasonable to consider immediate combination therapy with bempedoic acid.

Bempedoic acid is a prodrug that is orally administered once a day. It is rapidly converted in the liver to its active metabolite, which is associated with a lower risk of muscular adverse events compared to statins [20] and acts by inhibiting ATP citrate lyase. The CLEAR study (Cholesterol Lowering via Bempedoic Acid, an Adenosine Triphosphate Citrate Lyase [ACL]-Inhibiting Regimen) evaluated the benefits of bempedoic acid in terms of LDL reduction. The results demonstrated that a dosage of 180 mg of bempedoic acid, in addition to maximally tolerated statin therapy, led to an additional 15–20% reduction in plasma LDL levels. The impact of bempedoic acid on cardiovascular events is under assessment in the CLEAR Outcomes trial (NCT02993406), which recently completed the enrollment of 14,014 statin-intolerant, high-cardiovascular-risk, or known-cardiovascular-disease patients and has reported positive outcomes in reducing adverse cardiovascular events [21,22]. However, specific data regarding the impact of bempedoic acid on LDL reduction and its clinical effects after a recent acute coronary syndrome are currently unavailable, as patients of this type have been excluded from the trials conducted so far.

### 2.1. Atherosclerotic Plaque: Reduced Progression, Regression, and Stabilization

The pathogenetic mechanisms contributing to the formation and progression of atherosclerotic plaques are diverse. Furthermore, the underlying process is highly dynamic and associated with different phenomena [23]. At present, intracoronary imaging techniques allow for the definition of the extent and composition of atherosclerotic plaques in vivo, enabling the assessment of qualitative and quantitative variations induced by pharmacological therapy.

In a comparative study between atorvastatin (high-intensity regimen) and pravastatin (moderate regimen) [24], patients treated with atorvastatin demonstrated a lower progression of plaque compared to the cohort treated with pravastatin.

Several studies conducted in recent years [25] have also shown that a lipid-lowering drug not only slowed down the progression of coronary atherosclerotic plaques, but also induced significant regression. A recent trial [26] compared two statins at maximum dosage (i.e., rosuvastatin 40 mg/day and atorvastatin 80 mg/day) in patients with coronary artery disease. Both groups showed a substantial and significant regression of coronary atherosclerosis. Both treatment regimens induced regression in the majority of patients and had acceptable side-effect profiles, with a low incidence of laboratory abnormalities and cardiovascular events.

The effects of the combination of ezetimibe and atorvastatin on plaque regression have also been assessed [27]. The results indicated that combination therapy, in addition to achieving a greater reduction in LDL levels, was associated with a higher prevalence of regression.

The study by Banach et al. compared statin monotherapy and initial combination therapy with statin and ezetimibe in patients with acute coronary syndrome (ACS). The initial combination lipid-lowering therapy demonstrated superiority over statin monotherapy in reducing all-cause mortality among patients with ACS. These findings indicate that in high-risk patients, such a strategy should be recommended instead of a stepwise therapy approach [28].

In recent years, PCSK9 monoclonal antibodies have been widely used, in combination with statins and ezetimibe, in the treatment of patients with coronary artery disease. PCSK9 is a protein involved in regulating the degradation of LDL receptors on the cellular membranes of hepatocytes. Recent data suggest that circulating levels of PCSK9 increase within hours of the onset of acute coronary syndrome (SCA) and are associated with increased platelet anti-aggregation, the vulnerability of coronary plaque, elevated inflammatory markers, and higher long-term cardiovascular events [29]. Various evidence suggests that PCSK9 exerts deleterious effects on coronary plaques through different mechanisms; for example, in coronary plaque, inhibiting PCSK9 reduces the number of macrophages and the content of the necrotic core while promoting apoptosis [30]. Several trials have studied the in vivo effects of these monoclonal antibodies on atherosclerotic plaques. It is interesting to note that a clear correspondence between atherosclerosis regression and the clinical benefit of the molecule has been observed [31]. The monoclonal antibodies alirocumab and evolocumab, which are administered subcutaneously once or twice a month, have been shown to selectively inhibit the PCSK9 protein [32,33,34].

Another way to reduce PCSK9 levels is by inhibiting its gene expression through neutralizing its mRNA using short interfering RNA (siRNA) molecules. Inclisiran is a double-stranded ribonucleic acid that exerts a prolonged effect, reducing PCSK9 synthesis in hepatocytes. This medication is administered once every six months. In the recent ORION-10 (Inclisiran for Participants With Atherosclerotic Cardiovascular Disease and Elevated Low-Density Lipoprotein Cholesterol) and ORION-11 (Inclisiran for Subjects With ASCVD or ASCVD-Risk Equivalents and Elevated Low-Density Lipoprotein Cholesterol) studies, inclisiran was compared to a placebo in patients with known coronary artery disease (CAD) or equivalent risk factors and elevated levels of LDL cholesterol (≥70 or ≥100 mg/dL, respectively). In comparison to the placebo, inclisiran halved LDL cholesterol levels without significant safety differences [35]. The VICTORION-INCEPTION study (NCT04873934), with results expected in 2024, is assessing the effectiveness of early inclisiran administration in approximately 380 patients with recent acute coronary syndrome (within 5 weeks) and LDL cholesterol ≥ 70 mg/dL, in terms of the percentage reduction of LDL cholesterol and the achievement of lipid targets at 1-year follow-up, compared to standard therapy.

Another study, Evolocumab Very Early After Myocardial Infarction (EVOLVE-MI, NCT05284747), had the primary objective of evaluating the effectiveness of early treatment with evolocumab plus routine lipid management, compared to routine lipid management alone, when administered in the acute setting to reduce myocardial infarction, ischemic stroke, arterial revascularization, and death from all causes in subjects hospitalized for an acute myocardial infarction (non-ST-segment elevation myocardial infarction, NSTEMI; and ST-segment elevation myocardial infarction, STEMI). The results of the study are expected to be published in 2027.

Regarding the evaluation of plaque stabilization, numerous trials have been conducted to demonstrate the positive effects of lipid-lowering drugs. In one trial [36], the non-culprit coronary arteries of 300 patients were studied using intracoronary imaging, both upon admission and after 52 weeks. Compared to the group treated with high-intensity statin therapy alone, patients treated with alirocumab and statins showed a greater reduction in plaque volume and lipid burden in non-culprit arteries.

### 2.2. Lp(a)

The Lp(a) particle resembles LDL, being cholesterol-rich, with apolipoprotein B covalently linked to apolipoprotein(a) [apo(a)]. Its relationships with cardiovascular risk, atherosclerosis and coronary artery disease have received increasing attention [37,38]. Lp(a) has been associated with an increased risk of coronary and cerebrovascular events, peripheral artery disease, heart failure, and atrial fibrillation [39]. The Copenhagen City Heart Study, involving 9330 individuals, demonstrated a progressive increase in the risk of myocardial infarction with increasing Lp(a) levels over a 10-year follow-up, with no evidence of a risk threshold [40]. A notable characteristic of Lp(a) is its association with premature atherosclerosis and juvenile acute coronary syndrome (ACS). This relationship was assessed in a case–control study of 1457 ACS patients, showing that a 10 mg/dL increase in Lp(a) levels was associated with a 4% higher probability of ACS in young individuals (<45 years), compared to a 2% probability in middle-aged individuals (45–60 years). A distinction in Lp(a) levels has recently been proposed, where <30 mg/dL (75 nmol/L) excludes an increased CV risk and levels between 30 and 50 mg/dL (75–125 nmol/L) represent a “gray zone” in terms of risk [39]. The effects of statins on Lp(a) remain controversial. Several studies have shown an increase in Lp(a) levels in statin-treated patients, although this seems to occur only in patients with small apo(a) phenotypes. However, statins are still necessary in patients with high Lp(a) levels, in order to reduce their overall risk through intense LDL lowering [39]. Niacin lowers circulating ApoB-containing particle levels through various mechanisms. The effect of PCSK9 inhibitors on Lp(a) levels in addition to niacin therapy has recently been hypothesized. The associated results showed a median Lp(a) percentage reduction of 15.3% in patients receiving niacin with PCSK9 inhibitors, with an absolute Lp(a) reduction of 9 mg/dL. However, this reduction does not result in a decrease in CV events, as has been demonstrated in two clinical trials (AIM-HIGH and HPS2-THRIVE) [41]. Furthermore, combination therapy with niacin and statins increases the risk of serious adverse effects (e.g., the onset of diabetes, musculoskeletal symptoms, infections, and bleeding). The use of PCSK9 inhibitors has been assessed in the ODYSSEY Outcomes study, in which alirocumab was compared with placebo in 18,924 patients who had experienced recent acute coronary syndromes and were already receiving optimized statin therapy. Among these patients, 4351 individuals (23.0%) had a screening or randomization LDL-C level of less than 70 mg/dL (with a median of 69.4 mg/dL and an interquartile range of 64.3–74.0 mg/dL), while 14,573 patients (77.0%) had LDL-C levels of 70 mg/dL or higher in both screening and randomization (with a median of 94.0 mg/dL and an interquartile range of 83.2–111.0 mg/dL). In individuals who have recently experienced acute coronary syndromes and have LDL-C levels close to 70 mg/dL while on optimized statin therapy, the inhibition of proprotein subtilisin/kexin type 9 (PCSK9) offers additional clinical advantages only when the concentration of lipoprotein(a) is at least mildly increased [42]. Another study was conducted with the aim of evaluating whether there were notable sex–time interactions in the response of lipoprotein(a) (Lp(a)) and low-density lipoprotein cholesterol (LDL-C) to treatment with PCSK9i Evolocumab in a real-world clinical setting. Over the study duration, there was a significant decrease in the absolute values of Lp(a) plasma concentrations observed across the entire cohort (*p*-value < 0.001) and among both sexes (*p*-value < 0.001 in men and *p*-value = 0.002 in women). However, no significant sex-related differences were found. The absolute plasma concentrations of LDL-C also significantly decreased over time in the entire cohort and among both sexes (*p*-value < 0.001 in all cases), with greater improvements noted in men compared to women. A statistically significant sex–time interaction was observed in LDL-C (all *p*-values < 0.05), whereas absolute changes in Lp(a) were not influenced by either sex or time (all *p*-values > 0.05). These findings partially support the existence of variations in response to PCSK9i treatment between men and women and underscore the importance of understanding the interplay between LDL-C and Lp(a) reduction in response to PCSK9i. Further investigations are warranted to elucidate whether these sex-related significant differences translate into clinically meaningful distinctions in the long-term risk of CVD [43]. Mipomersen is an antisense oligonucleotide (ASO) targeting ApoB mRNA, reducing its synthesis through inhibiting mRNA translation. It can reduce both LDL cholesterol and Lp(a) by 21–50%, but its side-effects limit its use in subjects with homozygous familial hypercholesterolemia, and it has not been approved for reducing Lp(a) [44]. Cholesterol ester transfer protein (CETP) inhibitors (e.g., dalcetrapib, anacetrapib, evacetrapib, and obicetrapib) are pharmacological agents that increase HDL cholesterol levels. Their mechanism of action involves interfering with the exchange of cholesterol esters and triglycerides between HDL and LDL lipoproteins. Anacetrapib has been shown to reduce Lp(a) levels by 34.1%, primarily through reducing apo(a) production [45]. The reductions obtained with dalcetrapib and torcetrapib were considerably lower (5–10%) [46], while no data exist for the newest class member (obicetrapib). Except for the latter (which is still in development), none of the previous (not yet commercially available) drugs have substantially demonstrated a reduction in CV events, despite the increases in HDL cholesterol that they induce. Particularly promising data seem to emerge with the use of ASOs directed against apo(a) mRNA, specifically those reducing plasma Lp(a) levels. Pelacarsen is an ASO specific for apo(a) administered monthly with selective action at the hepatic level (determined through conjugation with triantennary N-acetylgalactosamine), where it binds to apo(a) mRNA, causing its degradation and a consequent inhibition of synthesis. Olpasiran (AMG890) is the more advanced siRNA that is specific for Lp(a). A phase 2 study [OCEAN(a)-DOSE; NCT04270760] that was recently published reported a 94% reduction with a dose of 75 mg every 3 months in 281 subjects with previous CV events. With a 225 mg dose, the reduction reached 98%, without serious safety issues detected. The phase 3 study (NCT05581303), which began enrollment in July of 2023, aims to include 6000 total patients with Lp(a) > 90 mg/dL to assess its efficacy in reducing CV events. Finally, a phase 1 trial of a new orally administered drug, muvalaplin, has recently been completed, with the results presented at the ESC Congress 2023. Muvalaplin is a small molecule capable of interrupting the initial non-covalent interaction between apo(a) and apoB, and it is the first orally administered drug developed to lower Lp(a) levels. In the phase 1 trial with human subjects who were healthy, muvalaplin was given orally in escalating single doses from 1 mg to 800 mg and in escalating multiple doses from 30 mg to 800 mg over a 14-day period. This led to dose-dependent increases in plasma concentration. There were no safety or tolerability issues associated with muvalaplin administration, and it resulted in decreased Lp(a) levels without affecting plasminogen activity [47].

## 3. Adherence to Treatment

It must be considered that real-world observations attest to a wide gap in the actual achievement of the targets recommended in the relevant guidelines. In the European observational DA VINCI study, 18% of 2888 secondary prevention patients had an LDL-C level < 55 mg/dL, and 45.8% of these were being treated with a low- or moderate-intensity statins [13]. The results of the EUROASPIRE IV study regarding lipid-lowering therapy in patients with coronary artery disease also demonstrated that, despite evidence of the benefits of intensive lipid-lowering therapy, many patients with coronary artery disease and dyslipidemia are not adequately treated (only one third of patients are discharged with intensive statin therapy).

This could be explained, on one hand, as being due to the under-utilization of combination therapies and, on the other hand, the low adherence to treatment, which is attributable, among various factors [48], to polypharmacological therapy itself, requiring patients with multiple comorbidities to take multiple medications [49].

A useful strategy to promote therapeutic adherence is the use of a single pill containing various pharmacological agents targeting the control of multiple risk factors or disease mechanisms [50]. A systematic literature review including nine randomized clinical trials highlighted that a polypill containing at least one antihypertensive and one lipid-lowering agent was associated with 33% higher therapeutic adherence compared to standard therapy [51]. At present, the European Society of Cardiology guidelines on the management of dyslipidemias recommend the use of medications containing multiple therapeutic agents in a single tablet. The impact of polypharmacology on therapeutic adherence has been further confirmed through the demonstration that patients on lipid-lowering therapy receiving the combination of statin + ezetimibe in a single pill had an 87% higher probability of being highly adherent to treatment, compared to those taking the two drugs separately, regardless of age, sex, or clinical profile, with a 55% reduction in cardiovascular outcomes compared to patients with low adherence [52,53].

It is evident that, in dyslipidemic patients at risk of developing cardiovascular pathologies, it is advisable to apply intensive lipid-lowering treatment while ensuring maximum therapeutic adherence. A recent study has evaluated the joint contribution of treatment intensity and adherence to prescribed therapy on cardiovascular events in patients with dyslipidemia and established cardiovascular risk factors or diseases, in which the association of both factors with a reduction in cardiovascular risk was confirmed, mediated by reduced LDL levels. The lowest cardiovascular risk was observed in patients with high adherence to the intensive lipid-lowering treatment.

## 4. Conclusions

Lipid-lowering therapy has been shown to be effective in preventing cardiovascular disease; however, reluctance to prescribe an intensive regimen and limited adherence to treatment still pose significant barriers to therapeutic optimization. Considering the ongoing high epidemiological impact of cardiovascular diseases and high mortality rates globally, it is essential to implement treatment strategies through the use of pharmacological combinations (e.g., moderate-intensity statins + ezetimibe) to improve therapeutic adherence and reduce the burden of atherosclerotic risk.

## Figures and Tables

**Table 1:** Categories of cardiovascular (CV) risk estimated with SCORE2/SCORE2-OP.

	<50 y.o.	50–69 y.o.	≥70 y.o.
Moderate–Low CV Risk: The treatment of risk factors is not generally recommended.	<2.5%	<5%	<7.5%
High CV Risk: The treatment of risk factors should be considered.	2.5–7.5%	5–10%	7.5–15%
Very High CV Risk: The treatment of risk factors is generally recommended.	≥7.5%	≥10%	≥15%

y.o.—Years old.

**Table 2:** Therapeutic targets according to ESC/EAS 2019 guidelines for the treatment of dyslipidemias.

Patients at Very High Risk in Secondary or Primary Prevention	<1.4 mmol/L (Less than 55 mg/dL) and a Reduction of ≥50% Compared to Baseline. For Patients with Atherosclerotic Cardiovascular Disease Experiencing a Second Event within 2 Years Despite Statin Therapy at the Maximum Tolerated Dose, a Target LDL Level of <1 mmol/L (<40 mg/dL) Could be Considered.
Patients at high cardiovascular risk	<1.8 mmol/L (less than 70 mg/dL) and a reduction of ≥50% compared to baseline.
Patients with moderate cardiovascular risk	<2.6 mmol/L (<100 mg/dL).

**Table 3:** Expected percentage reduction in LDL cholesterol levels.

Lipid-Lowering Treatment Intensity	
Treatment	Average Reduction in LDL
Moderate-intensity statin	≈30%
High-intensity statin	≈50%
High-intensity statin + ezetimibe	≈65%
PCSK9 inhibitor	≈60%
PCSK9 inhibitor + high-intensity statin	≈75%
PCSK9 inhibitor + high-intensity statin + ezetimibe	≈85%
Bempedoic acid/ezetimibe	≈33%
Bempedoic acid + statins at the maximum tolerated dose	≈68–78%
